# PUMA and NOXA Expression in Tumor-Associated Benign Prostatic Epithelial Cells Are Predictive of Prostate Cancer Biochemical Recurrence

**DOI:** 10.3390/cancers12113187

**Published:** 2020-10-29

**Authors:** Sylvie Clairefond, Benjamin Péant, Véronique Ouellet, Véronique Barrès, Zhe Tian, Dominique Trudel, Pierre I. Karakiewicz, Anne-Marie Mes-Masson, Fred Saad

**Affiliations:** 1 Centre de Recherche du Centre Hospitalier de L’Université de Montréal (CRCHUM) et Institut du Cancer de Montréal (ICM), Montreal, QC H2X 0A9, Canada; sylvie.clairefond@umontreal.ca (S.C.); benjamin.peant.chum@ssss.gouv.qc.ca (B.P.); veronique.ouellet.chum@ssss.gouv.qc.ca (V.O.); veronique.barres.chum@ssss.gouv.qc.ca (V.B.); zhe.tian@mail.mcgill.ca (Z.T.); dominique.trudel.chum@ssss.gouv.qc.ca (D.T.); pierre.karakiewicz@umontreal.ca (P.I.K.); anne-marie.mes-masson@umontreal.ca (A.-M.M.-M.); 2 Département de Médecine, Faculté de Médecine, Université de Montréal, Montreal, QC H3T 1J4, Canada; 3 Département de Pathologie et Biologie Cellulaire, Faculté de Médecine, Université de Montréal, Montreal, QC H3T 1J4, Canada; 4 Département de Chirurgie, Faculté de Médecine, Université de Montréal, Montreal, QC H3C 3J7, Canada

**Keywords:** prostate cancer, predictive biomarkers, biochemical recurrence, immunofluorescence, benign glands

## Abstract

**Simple Summary:**

After receiving a diagnosis of prostate cancer, patients follow a routine treatment plan based on tumor grade, stage and prostate-specific antigen (PSA) level. However, studies in other cancers have shown the importance of using biomarkers to personalize treatments. With no approved biomarkers presently in use in prostate cancer, there is a clinical need to develop such stratification tools. Here our study shows that PUMA and NOXA are markers that have a high prognostic value when looking at their presence in both benign and tumor glands within the prostate. Hence, the presence of these markers may help to better predict outcomes at diagnosis. Incorporating these markers into clinical practice may eventually lead to selective treatment options in newly diagnosed patients. This in turn should lead to better cancer control, potentially lowering the morbidity and mortality due to prostate cancer.

**Abstract:**

Background: Given that treatment decisions in prostate cancer (PC) are often based on risk, there remains a need to find clinically relevant prognostic biomarkers to stratify PC patients. We evaluated PUMA and NOXA expression in benign and tumor regions of the prostate using immunofluorescence techniques and determined their prognostic significance in PC. Methods: PUMA and NOXA expression levels were quantified on six tissue microarrays (TMAs) generated from radical prostatectomy samples (*n* = 285). TMAs were constructed using two cores of benign tissue and two cores of tumor tissue from each patient. Association between biomarker expression and biochemical recurrence (BCR) at 3 years was established using log-rank (LR) and multivariate Cox regression analyses. Results: Kaplan–Meier analysis showed a significant association between BCR and extreme levels (low or high) of PUMA expression in benign epithelial cells (LR = 8.831, *p* = 0.003). Further analysis revealed a significant association between high NOXA expression in benign epithelial cells and BCR (LR = 14.854, *p* < 0.001). The combination of extreme PUMA and high NOXA expression identified patients with the highest risk of BCR (LR = 16.778, *p* < 0.001) in Kaplan–Meier and in a multivariate Cox regression analyses (HR: 2.935 (1.645–5.236), *p* < 0.001). Conclusions: The combination of PUMA and NOXA protein expression in benign epithelial cells was predictive of recurrence following radical prostatectomy and was independent of PSA at diagnosis, Gleason score and pathologic stage.

## 1. Introduction

Prostate cancer (PC) remains one of the most common and lethal cancers in men worldwide. PC encompasses both low-risk disease that is slow growing and non-aggressive, and high-risk disease characterized by rapid progression and development of distant metastases [1]. Approximately a quarter of patients diagnosed with early stage PC will harbor high-risk disease. Although prostate-specific antigen (PSA) is used as a tumor marker to screen for PC [2], this has led to an increased incidence rate of PC and possible overtreatment of low-risk disease [2]. Currently, clinicians use tumor grade, tumor volume and clinical stage to determine prognosis and guide treatment decisions [3,4,5]. However, these clinical parameters are still imperfect in distinguishing between low and high-risk diseases. New predictive biomarkers are needed to complement available clinical parameters to improve patient management and outcome.

PUMA and NOXA are pro-apoptotic proteins in the BH3-only subgroup of the BCL2 family and are involved in the activation of the p53-dependent apoptosis pathway [6,7,8]. These proteins play a key role in the inhibition of pro-survival proteins of the BCL2 family (i.e., BCL2, BCL-XL or MCL1) [9,10] that drive the activation of pro-apoptotic activators such as BAX and BAK [11] that initiate the caspase cascade [11,12].

PUMA and NOXA have demonstrated a predictive potential in several cancers such as in hepatocellular carcinoma [13,14] and gallbladder adenocarcinoma [15,16], although their value in PC has not been fully elucidated. We previously reported on a small cohort of patients that expression of PUMA and NOXA is associated with PC progression [17]. In this study, we focused on the evaluation of PUMA and NOXA protein expression in a larger cohort of RP specimens. We optimized an immunofluorescence (IF) approach to allow proper digital quantification of protein levels to determine if PUMA and NOXA could be useful as PC biomarkers.

## 2. Results

### 2.1. Patient Characteristics and Clinical Parameters

The TF123 TMA series contains a non-selected retrospective cohort of patients (*n* = 300). Following extensive clinical data review, 15 were excluded from the analysis. Among the 285 eligible patients, PUMA and NOXA staining could be evaluated on benign tissues from 277 patients. None of the patients received neo-adjuvant androgen deprivation therapy prior to RP and median follow-up was 129 months. The incidence of BCR at 3 years was 27.3% (78 patients). Patient characteristics and clinical parameters are detailed in Table 1.

### 2.2. Multiplex Staining of TMA Cores

The basal expression of PUMA and NOXA proteins in PC cells was determined by western blots on whole cell extracts and by IF on a TMA containing formalin-fixed paraffin-embedded (FFPE) cell pellets of these PC cell lines (Figure S1 and Figure S5).

**Figure 1:**
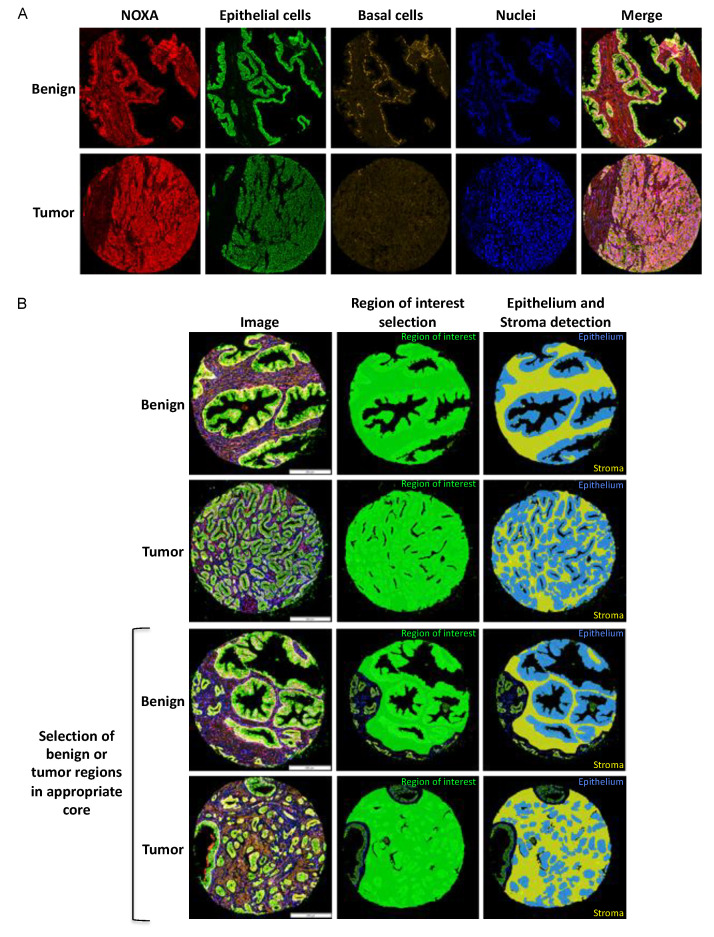
Digital imaging protocol for biomarker analyses. (**A**) Multiplex staining of TMA cores (benign and tumor) to discriminate biomarker expression (NOXA, red), epithelial luminal cells (cytokeratin CK8/18, green), basal cells (p63/CK HMW, yellow) and nuclei (DAPI, blue). Merge: superimposed images. (**B**) Representative analyses of biomarker detection in different cellular compartments using VisiomorphDP software. Images in the first column represent cores after IF staining. The second and third columns illustrate the use of specific compartmental algorithms within VisiomorphDP software: region of interest (green), epithelium (blue) and stroma (yellow). For each sub-compartment, the MFI for each specific biomarker was calculated.

To characterize the localization of PUMA and NOXA expression in various tissue and cellular compartments, a multi-staining IF approach was performed on TMAs using a cocktail-based strategy incorporating antibodies used to define specific regions for analysis (CK8/18, p63 and high molecular weight cytokeratin; CK HMW) as well as antibodies against NOXA or PUMA in conjunction with a nuclear mask (DAPI) (Figure 1A). The use of a digital image protocol allowed for the selection of different regions of interest using mask algorithms [18]. Basal cell markers (p63 and CK HMW) helped to differentiate between benign and tumor tissues which facilitates the analysis of regions of interest within each core (benign or tumor). After a manual exclusion of non-appropriate regions, a second algorithm based on the epithelial mask (cytokeratin 8 and 18, CK8/18) allowed the separate analysis of stromal and epithelial compartments (luminal and basal cells, Figure 1B).

**Figure 2:**
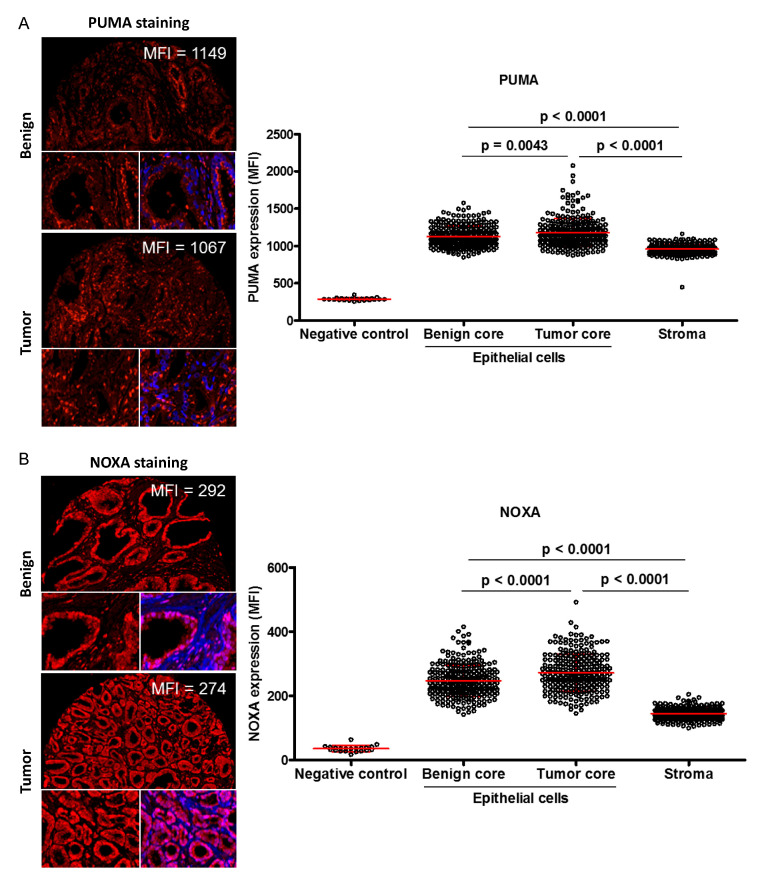
Compartment distribution of PUMA and NOXA expression in the prostate cancer (PC) patient cores. (**A**) Illustration of staining intensities (MFI) quantified by VisiomorphDP software was shown for moderate PUMA expression in one representative core of benign or tumor tissue. (**B**) Illustration of staining intensities (MFI) quantified by VisiomorphDP software is shown for moderate NOXA expression in one representative core of benign or tumor tissue. For each patient, expression levels were calculated using the average mean of two cores. Moderate staining was close to the median MFI intensity. Negative controls correspond to the quantification of MFI in cores when IF was performed with only secondary antibodies. Biomarker of interest (red), nuclei (blue) and merge (DAPI + marker) (purple).

PUMA expression was only observed in the cytoplasm of epithelial cells (Figure 2A and Figure S2A), whereas NOXA was found in both cytoplasmic and nucleic regions within epithelial cells (Figure 2B and Figure S2B). Both biomarkers were also detected in the stromal compartment. In the absence of specific markers for the stromal compartment and due to heterogeneity of the samples, data from tumor and benign cores from the same patient were pooled before analyses. Using specific settings for each biomarker, a VisiomorphDP algorithm calculated the individual MFI for PUMA and NOXA staining. For Gaussian distribution, the MFI of the protein of interest within the epithelial compartment (including benign and tumor values) was increased by 2.5-fold for PUMA (from MFI = 848 to MFI = 2078) and 3.5-fold for NOXA (from MFI = 141 to MFI = 492) (Figure 2A,B, respectively). PUMA (average MFI = 1154 for epithelial cells and 958 for stromal) and NOXA (average MFI = 259 for epithelial cells and 144 for stromal) staining intensity was significantly stronger in epithelial cells compared to the stromal compartment (*p* < 0.0001). Even if the tumor seemed significantly increased compared to benign glands for PUMA (*p* = 0.0043, average MFI = 1129 for benign and 1180 for tumor) and NOXA (*p* < 0.0001, average MFI = 247 for benign and 272 for tumor), the differences between each average expression were very similar (Figure 2A,B, respectively).

### 2.3. Expression of PUMA and NOXA Can Independently Predict BCR

**Figure 3:**
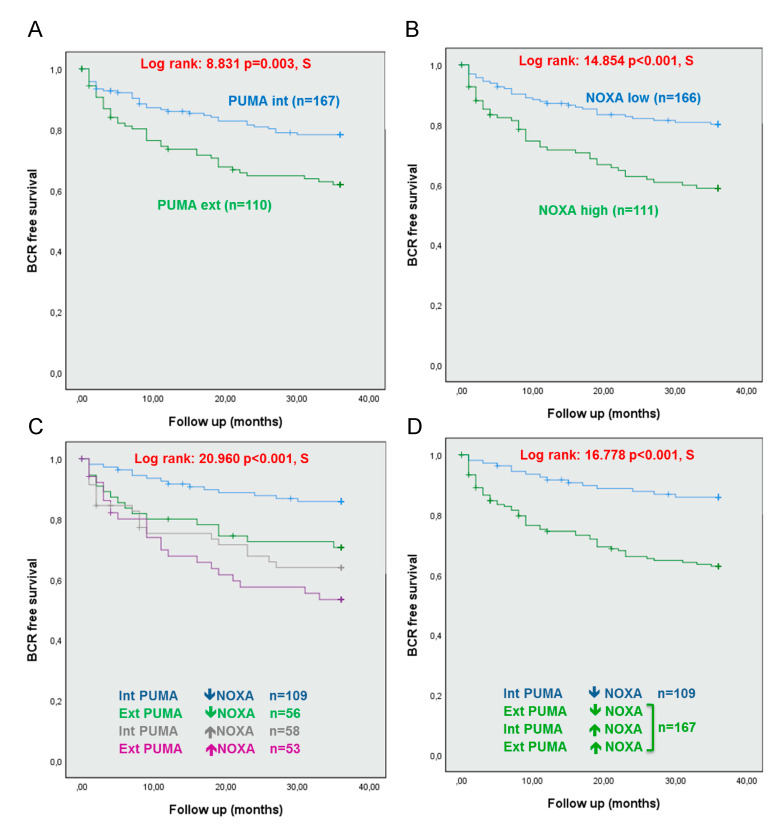
PUMA or NOXA expression in benign epithelium, alone or in combination, are associated with increased BCR risk at 3 years. Kaplan–Meir plots showing that (**A**) extreme expression of PUMA was associated to an increased risk of BCR at 3 years, (**B**) high NOXA expression was associated to an increased risk of BCR at 3 years, (**C**) combined expression of PUMA and NOXA in four groups was associated with BCR in PC patients at 3 years and (**D**) combined expression of PUMA and NOXA in two groups was associated with BCR in PC patients at 3 years. PUMA extreme (Ext) expression was <1st quintile and ≥5th quintile of MFI; PUMA intermediate (Int) expression was between ≥1st quintile and <5th quintile of MFI. NOXA high expression was ≥4th quintile and low expression was <4th quintile of MFI. A *p*-value < 0.05 was considered statistically significant (S).

Based on our previous work on a small cohort of patients, expression of PUMA and NOXA was associated with increased risk of BCR [17]. The median was used to dichotomize PUMA and NOXA expression data for all tissue compartments (Figure S3A,B, respectively). Using the median, only NOXA expression in benign epithelial cells was significantly associated with BCR (log rank = 5.854, *p* = 0.016). Based on Heagerty’s variant to ROC-curve to produce the AUC of both PUMA and NOXA for BCR at 3 years. PUMA had an AUC of 0.53161, and NOXA had an AUC of 0.62 (data not shown). To refine our analysis, data were organized as quintiles to identify groupings most susceptible to predict BCR for each biomarker (Figure S4A,B). In benign epithelial cells, quintile analysis of PUMA expression revealed two groups that were associated with BCR: the upper (≥5th quintile) and lower (<1st quintile) groups defined as extreme expression PUMA (log rank = 12.544, *p* = 0.014). For NOXA expression, quintile analysis demonstrated that the two-upper group (≥4th quintiles) seems to be associated with BCR (log rank = 8.599, *p* = 0.072). However, no significant results were obtained in tumor cells or in the stroma for PUMA and NOXA expression (Figure S4A,B). Using these new cut-offs, extreme PUMA and high NOXA expression in benign epithelial cells were associated with high risk of BCR at 3 years (log rank = 8.831, *p* = 0.003 and log rank = 14.854, *p* < 0.001, respectively) (Figure 3A,B).

Based on these results, univariate Cox regression analysis was performed for these biomarkers in benign epithelial cells only (Table 2). PUMA or NOXA dichotomized by quintile data showed a strong association with increased risk of BCR (HR = 1.953, *p* = 0.004 for PUMA and HR = 2.377, *p* < 0.001 for NOXA) (Table 2). The multivariate Cox regression analysis included following clinical markers: PSA at time of diagnosis, Gleason score at RP, margin status and pathologic stage (pTNM). Dichotomized expression of PUMA and NOXA by quintile were found to be statistically significant in a multivariable model (HR = 2.173, *p* = 0.001 for PUMA and HR = 2.280, *p* = 0.001 for NOXA) (Table 2) and identified as two independent markers with better predictive capabilities than diagnostic PSA, Gleason score and pathologic stage (Table 2).

### 2.4. Combining Expression Levels of PUMA and NOXA in Benign Cells Can Predict BCR

Since PUMA and NOXA expression levels were independently predictive, we investigated their potential as combined biomarkers. Following dichotomization by quintiles, four groups were created to analyze the impact of PUMA and NOXA expression levels on BCR: intermediate PUMA with low NOXA (group 1, blue line); extreme PUMA with low NOXA (group 2, green line); intermediate PUMA with high NOXA (group 3, grey line); and extreme PUMA with high NOXA (group 4, purple line) (log rank = 20.960, *p* < 0.001, Figure 3C). Kaplan–Meier analyses showed that 3 groups presented a similar BCR risk. Hence, these three groups were merged together for subsequent analyses in which the combination of PUMA and NOXA expression consolidated their association with BCR at 3 years (log rank = 16.778, *p* < 0.001) (Figure 3D). Based on these results, univariate Cox regression analysis showed an increased BCR risk at 3 years when expression of PUMA and NOXA were combined (Table 3). This was also observed in multivariate Cox regression analysis (Table 3). More specifically, when there is an extreme PUMA or a high NOXA (HR = 2.935, *p* < 0.001), patients are at higher risk of BCR. This combination was a stronger predictor of BCR at 3 years than PSA at diagnosis, Gleason score, Margin and pTNM in multivariate analyses (Table 3).

Based on our Kaplan–Meier and Cox regression analyses, we propose a combination of PUMA and NOXA as biomarkers to discriminate patients at the time of radical prostatectomy. Patients with lower BCR risk have an intermediate PUMA expression coupled with low NOXA expression (15 with BCR of 109 patients, 14%) in the benign tissue. In contrast, patients with extreme PUMA expression and/or high NOXA expression (59 with BCR of 167 patients, 35%) are at higher risk of early BCR.

## 3. Discussion

Biomarker discovery and validation remains critical for improving personalized care in PC and for predicting outcomes in newly diagnosed patients. The necessity to discriminate patients who need aggressive therapy from those who can be safely managed by active surveillance continues to be a challenge and biomarkers that show utility in the pre-treatment setting would be particularly useful.

Diallo et al [17] showed that low expression levels of PUMA and NOXA in tumor cells were associated with a rapid progression towards BCR for PC patients; however, this result was reported for a small cohort of RP specimens (*n* = 62) of which half had positive surgical margins. Since positive surgical margins are a known risk factor for BCR, these particular patients may have enriched the cohort creating a bias that influenced the effect of PUMA and NOXA in that analysis. In our study, we used a larger cohort (*n* = 285) and improved the quantification of protein expression by IF through an automated calculation of the MFI for each biomarker to obtain a continuous value instead of using a scoring scale based on manual scoring (usually from 0 to 3) by immunohistochemistry. Our results demonstrate IF as a promising technique for biomarker quantification in a clinical setting as it uses small amounts of tissue, and digital scoring that ensures standardized, reproducible and rapid results. In addition, IF can include multiple staining for quantification of expression in specific compartments (epithelium/stroma/nuclei) and simultaneous analysis of different markers on the same tissue core. In contrast to our study, Diallo et al [17] only determined a link between expression of PUMA and NOXA and PC progression in tumor tissue, and did not investigate the expression of both biomarkers in benign tissue.

We identified the pro-apoptotic proteins PUMA and NOXA as potential biomarkers when expressed in benign epithelial cells that discriminate PC patients with an increased risk of BCR. This observation was independent of PSA at diagnosis, Gleason score and pathologic stage. In other tumors such as in hepatocellular carcinoma [13,14] and gallbladder adenocarcinoma [15,16], PUMA or NOXA expression was associated significantly with poorer prognosis. However, in several other tumor models the prognostic value of PUMA or NOXA was generally inconclusive [19,20,21,22,23].

Recently, studies have shown the importance of studying benign tissue adjacent to the tumor. The study by Bergstorm et al [24] suggest that the strong expression of microseminoprotein-beta (MSMB), a PC serum biomarker, in benign tissue was associated with higher tumor grade and aggressiveness. In addition, Yang et al [25] showed that an abnormal level of methylation in distant benign tissue from cancerous glands correlated with a more aggressive form of PC. Furthermore, Adamo et al [26] showed that tumor instructed normal tissue changes the volume and aggressiveness of tumors in prostate cancer. Together, these studies put into perspective the relevance of our study where the severity of the disease and patient outcome can potentially be determined with a biopsy specimen containing benign tissue.

## 4. Materials and Methods

### 4.1. Antibody Validation

PC cell lines 22Rv1, LNCaP, DU145 and PC3 were obtained from the American Type Culture Collection (ATCC, Manassas, VA, USA). Cells were maintained in RPMI 1640 medium (Wisent Inc., St-Bruno, QC, Canada) supplemented with 10% fetal bovine serum (FBS), 0.454 μg/mL amphotericin B and 90 μg/mL gentamycin (Gibco^®^, Thermo Fisher Scientific, Waltham, MA, USA).

### 4.2. Paraffin Processing and Embedding of Cell Line Pellets

PC cell lines were prepared as cell pellets, fixed and embedded in paraffin using HistoGel™ (Thermo Fisher Scientific) as previously described [27] and used for the construction of cell line microarrays with a high cell density per core.

### 4.3. siRNA Transfection

siRNAs targeting NOXA and siRNA control (siScramble) were obtained from Dharmacon Inc. (Lafayette, CO, USA). Transient transfections were performed on DU145 cells according to manufacturer’s instructions (Dharmacon Inc.). Briefly, for each siRNA, 1 × 10^6^ cells were suspended in a solution containing 90 μL of Nucleofector Solution V (Lonza, Basel, Switzerland), 6 μL of siRNA and 96 μL of RPMI 1640 medium. Transfection was performed using the Nucleofector Program X-005 in the Nucleofector Device and transfected cells were divided in 6-well plates. Two days after transfection, cells were collected for Western blot analysis. The following siRNA sequences were used in this study: siNOXA-1 AAACUGAACUUCCGGCAGA, siNOXA-2 GAACCUGACUGCAUCAAAA, siNOXA-3 AAUCUGAUAUCCAAACUCU and siNOXA-4 GCAAGAACGCUCAACCGAG.

### 4.4. Western Blot Analysis

Confluent cells were harvested and incubated 1 h at room temperature with lysis buffer (1% Triton, 10% glycerol, 50 mM Tris, 2 mM EDTA and 150 mM NaCl) supplemented with fresh protease inhibitors. After centrifugation, protein concentrations of whole cell extracts were determined by Bradford assay (Bio-Rad, Hercules, CA, USA). Proteins (30 μg) were separated on 15% sodium dodecyl sulphate polyacrylamide gel electrophoresis (SDS-PAGE, Bio-Rad) gel and transferred onto nitrocellulose membrane using the Trans-Blot Turbo Transfer System (Bio-Rad). The membrane was immunoblotted with either rabbit monoclonal anti-PUMA (1:1000, EP512Y, ab33906, Abcam Inc., Cambridge, UK) or mouse monoclonal anti-NOXA (1:250, 114C307.1, MA1-41000, Thermo Fisher Scientific) antibodies. Each primary antibody was diluted in Tris-buffered saline tween 20 (TBS-T) supplemented with 5% fat-free milk powder. β-actin was used as a loading control (AC-15, ab6276, Abcam Inc.). Immunoreactive bands were detected by enhanced chemiluminescence (ECL, GE Healthcare, Little Chalfont, UK). PUMA or NOXA signal quantification (relative to β-actin expression) was performed by Image Lab™ Version 6.0.0 (Bio-Rad).

### 4.5. Patient Cohort

Tissue microarray (TMA) TF123 is composed of tissue from 300 primary PC patients who underwent radical prostatectomy (RP) at the Centre hospitalier de l’Université de Montréal (CHUM, Montréal, QC, Canada) between 1993 and 2006. All subjects gave their informed consent in the PC biobank of the CHUM, affiliated to the Réseau de la recherche sur le cancer (RRCancer), for inclusion before they participated in the study. The study was conducted in accordance with the Declaration of Helsinki, and the protocol was approved by the Ethics Committee de la recherche du CHUM on 11 October 2012 (#2013-4072, CE 12.216-BSP). This study only included patients that were treatment naïve with at least 5 years of follow up following surgery. The cohort of patients from TMA TF1 contains an equal portion of patients with either a rapid (<1 year), intermediate (between 1 and 5 years) or no biochemical recurrence (BCR). The TF123 TMAs regroups randomly selected biobank participants between (1999–2006). The time to BCR was defined as the time interval between the date of the RP and the date of a PSA level above 0.2 ng/mL and rising. Gleason grade was extracted from diagnostic pathology reports according to ISUP 2005.

### 4.6. Construction of the Tissue Microarray

The prostate specimen was processed according to standard CHUM procedures. Based on pathology report, 2–3 blocks per patients were retrieved from the institutional archives and a fresh Hematoxylin and Eosin stained slide was reviewed by an expert genito-urinary pathologist. Tumor and benign non-malignant area were indicated on the Hematoxylin and Eosin section and regions containing inflammation, PIN and atrophy were excluded. The tumor core selected was based on primary Gleason grade. When feasible, the adjacent benign tissue was taken from the same block, however when tissue was scarce the benign region was taken from an adjacent paraffin block. Using the TMArray (Pathology Devices Inc., Westminster, MD, USA) two cores (0.6 mm) of both selected areas (adjacent benign and tumor) were randomly included on two receiver blocks (1 benign and 1 tumor core per block). The resulting TMA underwent a second review by a genito-urinary-pathologist. TMA blocks were sectioned at 4 µm TMA sections were used for subsequent IF assay [28].

### 4.7. Immunofluorescence

A semi-automatic IF protocol was used for multiplex staining. Briefly, TMA slides were deparaffinized and antigen retrieval using the Benchmark XT autostainer (VMSI). All antibodies and conditions used for this assay are summarized in Table S1 for primary antibodies and Table S2 for secondary antibodies. Primary antibodies were diluted in phosphate-buffered saline (PBS), added manually to slides and incubated at 37 °C for 60 min. The following manual steps were performed away from light. Slides were blocked with a protein block serum-free solution (DAKO, Agilent, Santa Clara, CA, USA) for 20 min. Secondary fluorescent antibodies were diluted in PBS containing 1% bovine serum albumin (BSA, Sigma-Aldrich, St-Louis, MO, USA), added to slides for a 45 min. incubation at room temperature. Slides were blocked overnight with 250 μL of PBS containing 50 μL of Mouse-On-Mouse (MOM) blocking reagent (MKB-2213, Vector Laboratories Inc., Burlingame, CA, USA). Antibody cocktails (CK8/18, p63 and high molecular weight cytokeratin; CK HMW) were used to detect and distinguish luminal epithelial cells and basal cells. All slides were stained with DAPI to identify nuclei. To quench tissue autofluorescence, each slide was incubated for 15 min. at room temperature with a 0.1% solution of Sudan Black B (Research organics, Cleveland, OH, USA) in 70% ethanol. Between each step, all slides were washed twice with 1X PBS. Slides were stored overnight at room temperature and scanned the following day. Negative control slides were performed in parallel (one for PUMA and one for NOXA) and incubated only with the corresponding secondary antibodies.

All slides were scanned with a 20× Olympus Optical microscope BX61VSF (Olympus, Shinjuku, Tokyo, Japan) and visualized with OlyVIA software (Olympus). Scanned images were imported to VisiomorphDP software (Visiopharm, Hoersholm, Denmark). This software used the multiplex staining for IF to develop fully functional and semi-automated Analysis Protocol Packages (APPs) to determine expression levels of each biomarker by the mean fluorescence intensity (MFI) in each defined compartment (i.e., stroma, epithelium cytoplasm) [18].

### 4.8. Statistical Analysis

Statistical analysis involved date pre-processing. First, cores damaged during the staining process were eliminated from analysis. Second, tumor and benign cores that contained less than 5% of epithelial cells were eliminated. Third, identification of duplicate cores with a significant standard deviation between them were performed by GraphPad Prism software V5 (GraphPad, La Jolla, CA, USA), and image control was done on VisiomorphDP software to evaluate if differences were related to technical issues, in which case the core was eliminated from the analysis. To compare biomarker expression between each compartment, the Mann-Whitney test was used. A mean fluorescence intensity (MFI) normalization compensated for the observed differences related to slide scanning and imaging in the average fluorescence intensity between each slide. Normalization to the MFI mean for each biomarker and in each compartment was achieved with the following equation example: (Mean of MFI_biomarker in epithelium (all slides of the series))/(Mean of MFI_biomarker in epithelium (slide of interest)) = Normalizing ratio. This normalizing ratio was applied to each biomarker score in the corresponding compartment: MFI_biomarker in epithelium (core of interest) × normalizing ratio = MFI_biomarker normalized in epithelium. After normalization and for each patient, the mean of the core MFI values (benign or tumor) was calculated prior to subsequent statistical analysis using SPSS Statistics 25.0 software package (SPSS Inc., Chicago, IL, USA). The data were first analysed using the median. Then they were analyzed as quintiles to explore data trends and identify the threshold. Survival curves plotted were established using the Kaplan-Meier method, and log-rank as well as Cox regression analyses tested for statistical significance in observed differences. Univariate and multivariate (Cox regression) analyses were used to estimate the hazard ratio (HR) for each biomarker. A two-sided *p*-value < 0.05 was considered statistically significant.

## 5. Conclusions

In this study, we identified two potential biomarkers in tumor-associated benign epithelial cells that discriminated high-risk patients with PC, independent of PSA at diagnosis, Gleason score, surgical margin status and pathologic stage. The clinical utility of biomarkers in benign tissue has significant potential for prognostic assessment of whole RP specimens and biopsies. Given the intrinsic sampling error of prostate biopsies, PUMA and NOXA expression levels in benign tissue may eventually serve as biomarkers to improve the identification of patients who are less suitable for active surveillance.

## Figures and Tables

**Table 1:** Clinical and pathological characteristics of the 285 patients who underwent radical prostatectomy.

Clinical Parameters	TF123 TMA Series
Median age at RP, years (IQR)	63 (59–67)
Median PSA at diagnosis, ng/mL (IQR)	7.0 (5.0–10.8)
Pathological TNM	
2	201
3	75
4	9
Gleason score at RP	
≤3 + 3	140
3 + 4	93
4 + 3	19
≥4 + 4	29
Unknown	4
Positive margin	95
Median follow-up, months (IQR)	129 (76–174)
Biochemical recurrence at 10 years	
No	177
Yes	108
Median time to BCR, months (IQR)	16 (4–41)
Biochemical recurrence at 3 years	
No	207
Yes	78
Median time to BCR, months (IQR)	8 (2–19)

Abbreviations: RP = radical prostatectomy; PSA = prostate-specific antigen; IQR = interquartile range; TNM = tumor, lymph nodes, metastasis; BCR = biochemical recurrence.

**Table 2:** Univariate and multivariate Cox regression analyses of clinical parameters and PUMA/NOXA biomarker expression in benign epithelial cells to predict biochemical recurrence at 3 years.

Clinical Parameters	Univariate			Multivariate with PUMA Expression			Multivariate with NOXA Expression		
	HR (95% CI)		*p*-Value	HR (95% CI)		*p*-Value	HR (95% CI)		*p*-Value
Age at Dx	1.015	(0.974–1.057)	0.482	−	−	−	−	−	−
PSA at Dx	1.048	(1.017–1.081)	0.003	1.014	(0.975–1.056)	0.483	1.029	(0.989–1.070)	0.160
Gleason score	1.809	(1.473–2.221)	0.000	1.547	(1.234–1.940)	0.000	1.552	(1.252–1.923)	0.000
Margin	3.890	(2.452–6.170)	0.000	2.567	(1.556–4.234)	0.000	2.458	(1.475–4.096)	0.001
cTNM (category)	1.031	(0.587–1.810)	0.916	−	−	−	−	−	−
pTNM (category)	2.934	(2.122–4.056)	0.000	1.957	(1.286–2.978)	0.002	1.672	(1.111–2.516)	0.014
PUMA (dichotomized)	1.953	(1.241–3.075)	0.004	2.173	(1.366–3.456)	0.001	−	−	−
NOXA (dichotomized)	2.377	(1.504–3.759)	0.000	−	−	−	2.280	(1.417–3.670)	0.001

Abbreviations: Dx = diagnosis; PSA = prostate-specific antigen; cTNM = clinical tumor, lymph nodes, metastasis; pTNM = pathological tumor, lymph nodes, metastasis; HR = hazard ratio; CI = confidence interval. Gleason score categorized: ≥ 3 + 3, 3 + 4, 4 + 3 or ≤ 4 + 4. pTNM (category): 2, 3 or 4. PUMA (dichotomized): extreme expression was <1st quintile and ≥5th quintile of MFI and intermediate expression was between 1st and 5th quintile of MFI. NOXA (dichotomized): high expression ≥4th quintile and low expression <4th quintile of MFI. Significant results (*p* < 0.05) are indicated by bold numbers and results not included are indicated by −.

**Table 3:** Univariate and multivariate Cox regression analyses of clinical parameters and combination of PUMA and NOXA expression in benign epithelial cells to predict biochemical recurrence at 3 years.

Clinical Parameters	Univariate			Multivariate		
	HR (95% CI)		*p*-Value	HR (95% CI)		*p*-Value
Age at Dx	1.015	(0.974–1.057)	0.482	−	−	−
PSA at Dx	1.048	(1.017–1.081)	0.003	1.022	(0.981–1.065)	0.293
Gleason score (category)	1.809	(1.473–2.221)	0.000	1.547	(1.240–1.931)	0.000
Margin	3.890	(2.452–6.170)	0.000	2.481	(1.484–4.148)	0.001
cTNM (category)	1.031	(0.587–1.810)	0.916	−	−	−
pTNM (category)	2.934	(2.122–4.056)	0.000	1.679	(1.109–2.543)	0.014
Combination of PUMA_ (dichotomized) and NOXA (dichotomized)	3.055	(1.732–5.386)	0.000	2.935	(1.645–5.236)	0.000

Abbreviations: Dx = diagnosis; PSA = prostate-specific antigen; cTNM = clinical tumor, lymph nodes, metastasis; pTNM = pathological tumor, lymph nodes, metastasis; HR = hazard ratio; CI = confidence interval. PUMA (dichotomized): extreme expression was <1st quintile and ≥5th quintile of MFI and intermediate expression was between 1st and 5th quintile of MFI. NOXA (dichotomized): high expression ≥4th quintile and low expression <4th quintile of MFI. Significant results (*p* < 0.05) are indicated by bold numbers and results not included are indicated by −.

## Supplementary Materials

The following are available online at https://www.mdpi.com/2072-6694/12/11/3187/s1, Figure S1: Validation of PUMA and NOXA antibody specificity in PC cell lines, Figure S2: Intensities of PUMA and NOXA staining in benign and tumor epithelial cells, Figure S3: Impact of PUMA or NOXA expression on patient risk of BCR evaluated by Kaplan–Meier analyses couples with a log-rank test, Figure S4: Analyses of PUMA and NOXA potential to predict BCR using quintile methods, Figure S5: Whole Western blots of PUMA and NOXA expression in PC cell lines, Table S1: Description of primary antibodies and conditions used for IF, Table S2: Description of secondary antibodies and conditions used for IF.
