# Supplementary material for: PUMA and NOXA Expression in Tumor-Associated Benign Prostatic Epithelial Cells Are Predictive of Prostate Cancer Biochemical Recurrence
Source: Cancers (Basel). 2020 Oct 29;12(11):3187. doi: 10.3390/cancers12113187 (PMC7692508; doi:10.3390/cancers12113187)
Supplement: Supplementary file 1 [file cancers-12-03187-s001.pdf]

Supplementary Materials:

PUMA and NOXA Expression in Tumor-Associated Benign Prostatic Epithelial Cells Are Predictive of Prostate Cancer Biochemical Recurrence

Sylvie Clairefond, Benjamin Péant, Véronique Ouellet, Véronique Barrès, Zhe Tian, Dominique Trudel, Pierre I. Karakiewicz, Anne-Marie Mes-Masson and Fred Saad

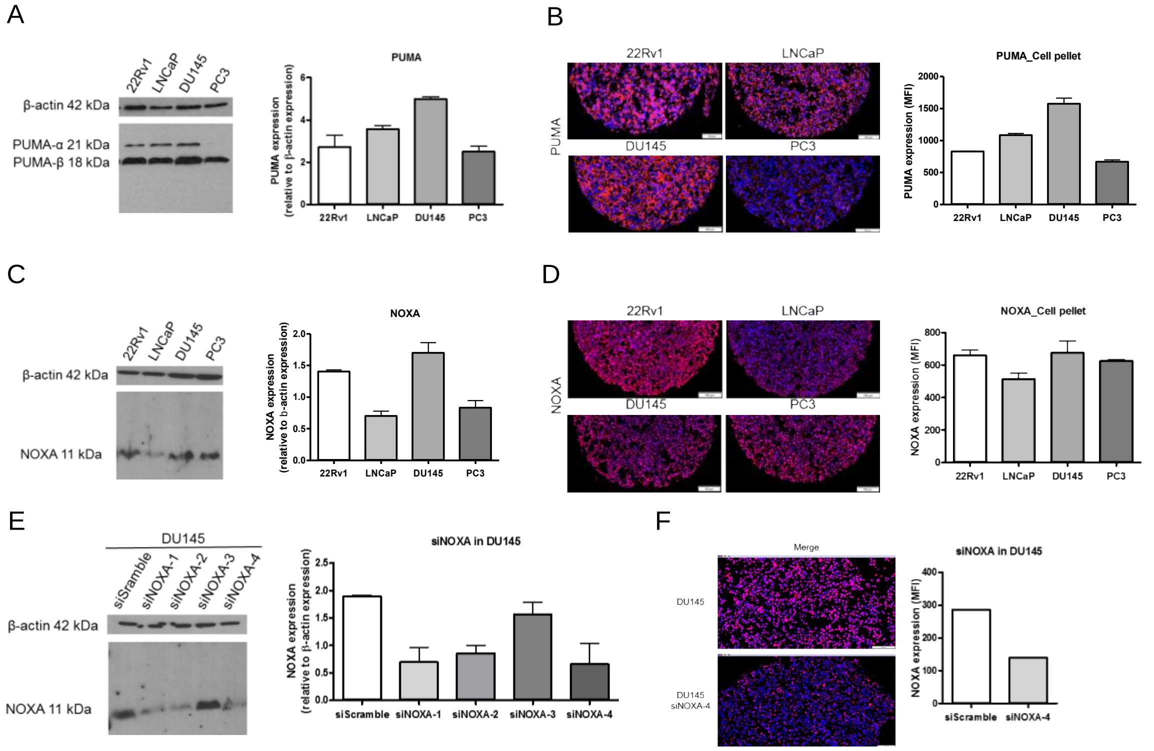

Figure S1. Validation of PUMA and NOXA antibody specificity in PC cell lines. (**A**) Expression of PUMA quantified in PC cell lines by western blot. The selected antibody detected only PUMA-α and PUMA-β (21 kDa and 18 kDa, respectively) [1,2]. All cell lines expressed both isoforms except PC3, which only expressed PUMA-β. PUMA (PUMA-α and PUMA-β) expression was higher in DU145 and LNCaP cell lines compared to 22Rv1 and PC3 cell lines. (**B**) Evaluation of PUMA expression detected in paraffin-embedded PC cell pellets by IF and quantification of staining intensities (MFI). The IF analysis (six pellet cores per cell line) of PUMA-α and PUMA-β showed relative expression patterns among cell pellets similar to those shown by western blot. (**C**) Expression of NOXA quantified in PC cell lines by western blot. NOXA expression was highest in DU145, followed by 22Rv1 and PC3 cell lines. LNCaP cell line expressed the lowest level of NOXA. (**D**) Evaluation of NOXA expression in FFPE PC cell pellets by IF. NOXA expression quantified in six different cores per cell line showed a relatively similar expression profile among DU145, 22Rv1 and PC3 cell lines. However, in LNCaP cells, NOXA expression levels were slightly different between western blot and IF results, and this difference could potentially be due to antibody specificity. To test antibody specificity, we introduced different anti-NOXA siRNAs to silence the NOXA protein coding gene in DU145. (**E**) The effects of four different anti-NOXA siRNAs were tested by western blot and their effects on NOXA expression was quantified. (**F**) siNOXA-4 was transfected in DU145 cells and NOXA expression was detected by IF. These results confirmed the specifity of NOXA antibody. β-actin served as loading control for quantification of western blots. Blue: nuclei, red: PUMA or NOXA. Scale bar: 100 μm.

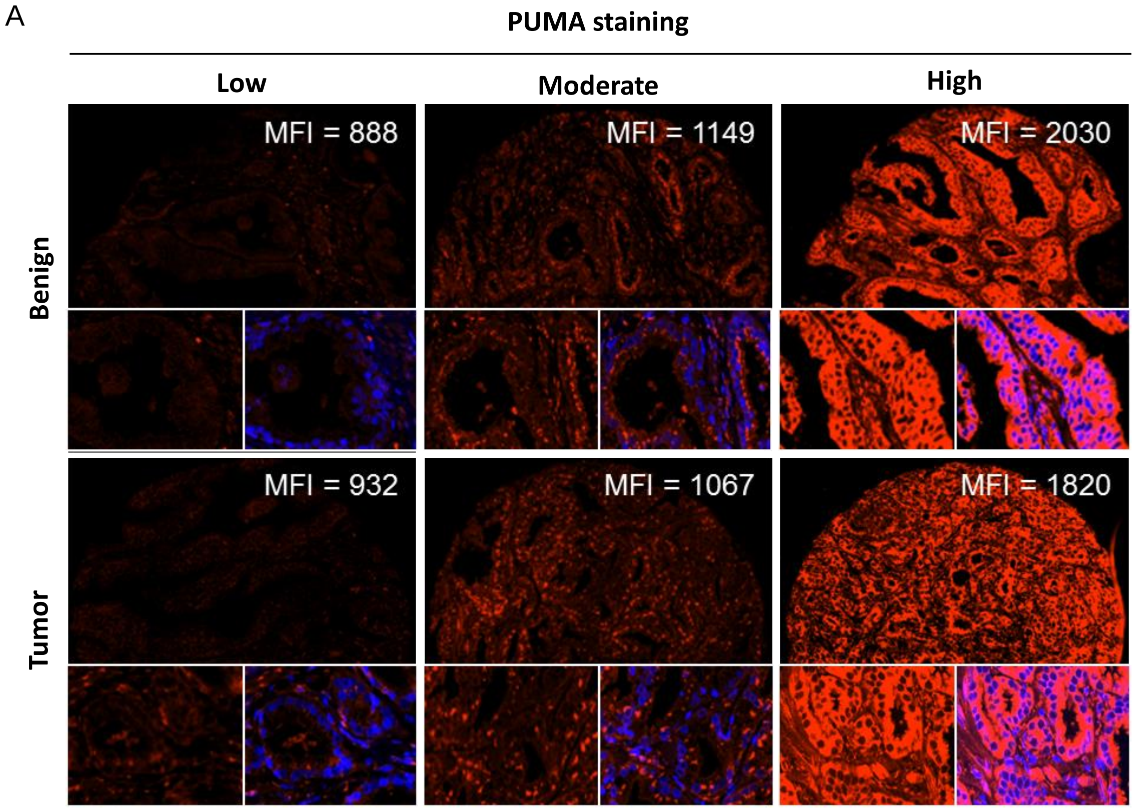

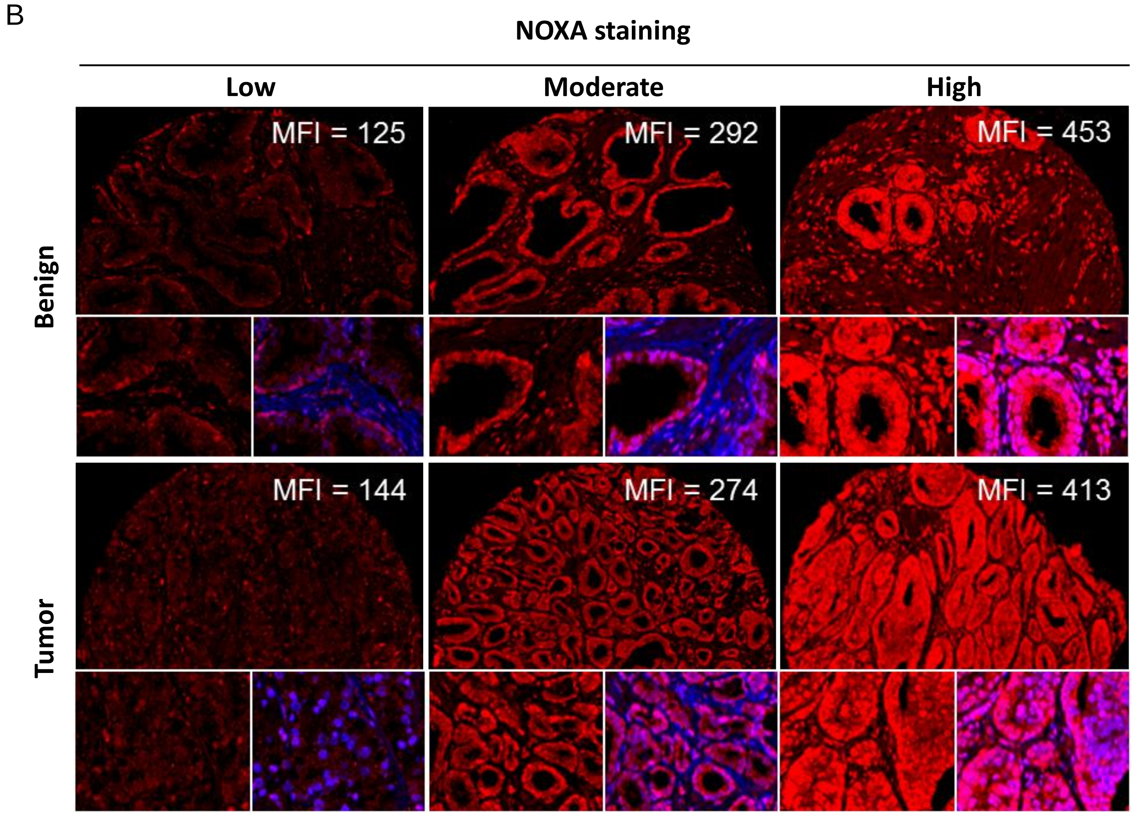

Figure S2. Intensities of PUMA and NOXA staining in benign and tumor epithelial cells. Staining intensities (low, moderate and high MFI) for (**A**) PUMA and (**B**) NOXA quantified by VisiomorphDP software are shown for each core. Low corresponded to 25th percentile intensity, moderate was close to median MFI intensity and high was representative of the 75th percentile intensity for PUMA or NOXA expression. PUMA or NOXA (red), nuclei (blue) and merge DAPI + marker (PUMA or NOXA) (purple).

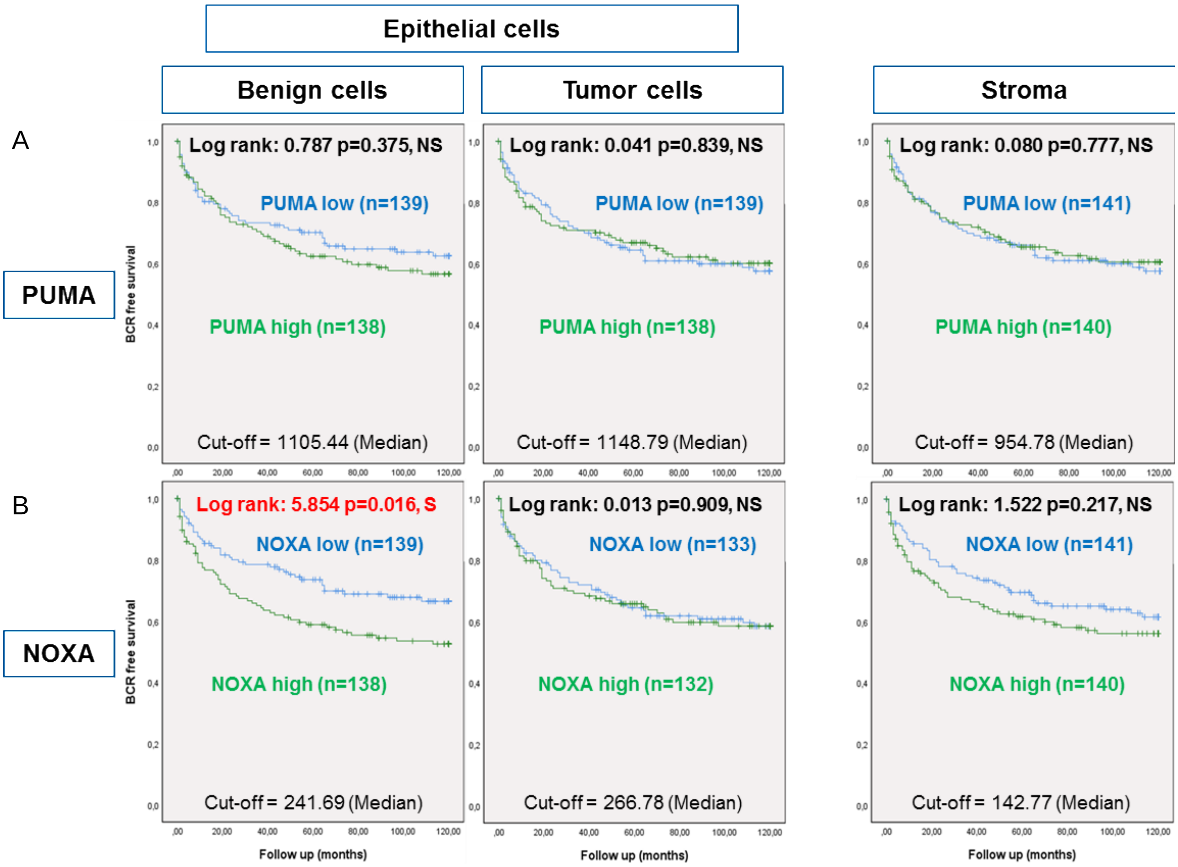

Figure S3. Impact of PUMA or NOXA expression on patient risk of BCR evaluated by Kaplan-Meier analyses coupled with a log-rank test. (**A**) High (over 50% of the median) and low (under 50% of the median) MFIs of PUMA in epithelial cells (benign and tumor) and in stroma. (**B**) High (over 50% of the median) and low (under 50% of the median) MFIs of NOXA in epithelial cells (benign and tumor) and in stroma. A *p*-value < 0.05 was considered statistically significant. NS: not significant. S: significant.

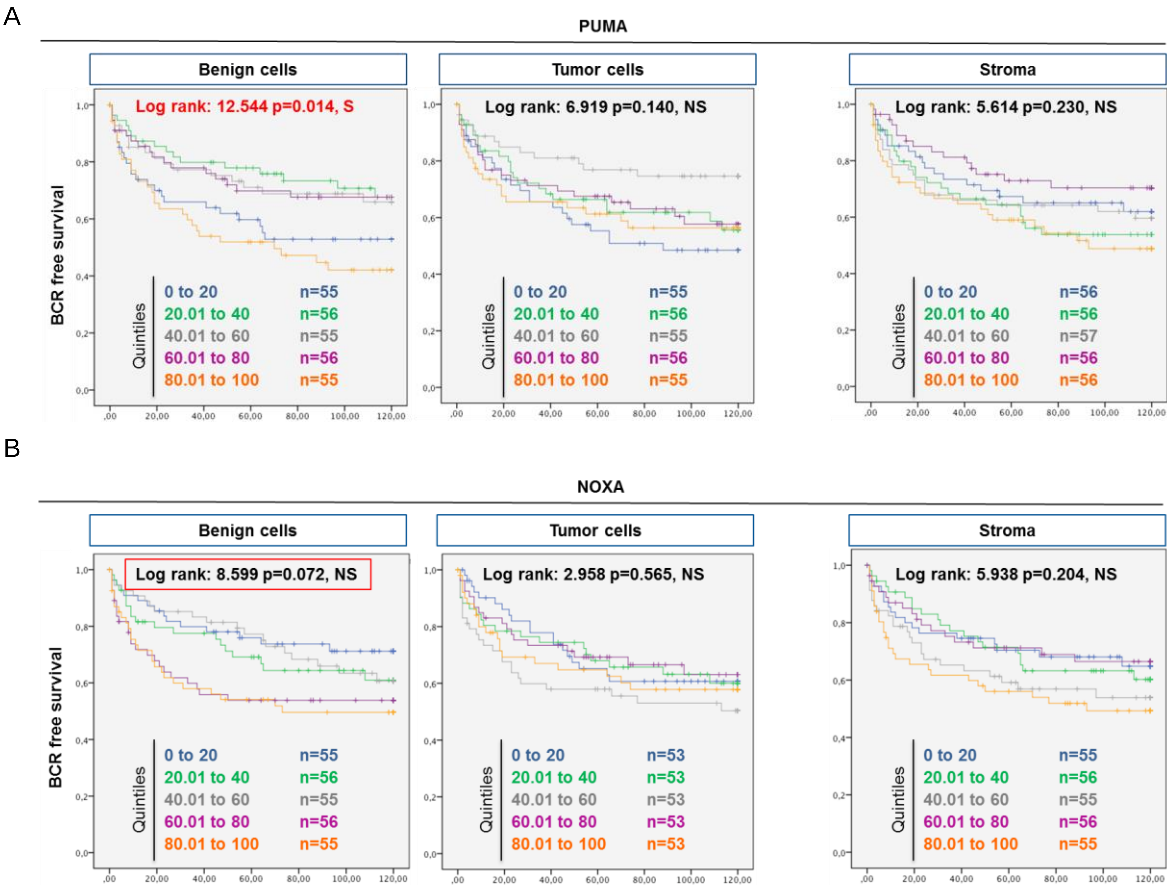

Figure S4. Analyses of PUMA and NOXA potential to predict BCR using quintile methods. Expression of (**A**) PUMA and (**B**) NOXA in benign and tumor epithelial cells, and stroma. A *p*-value < 0.05 was considered statistically significant. NS: not significant. S: significant.

A

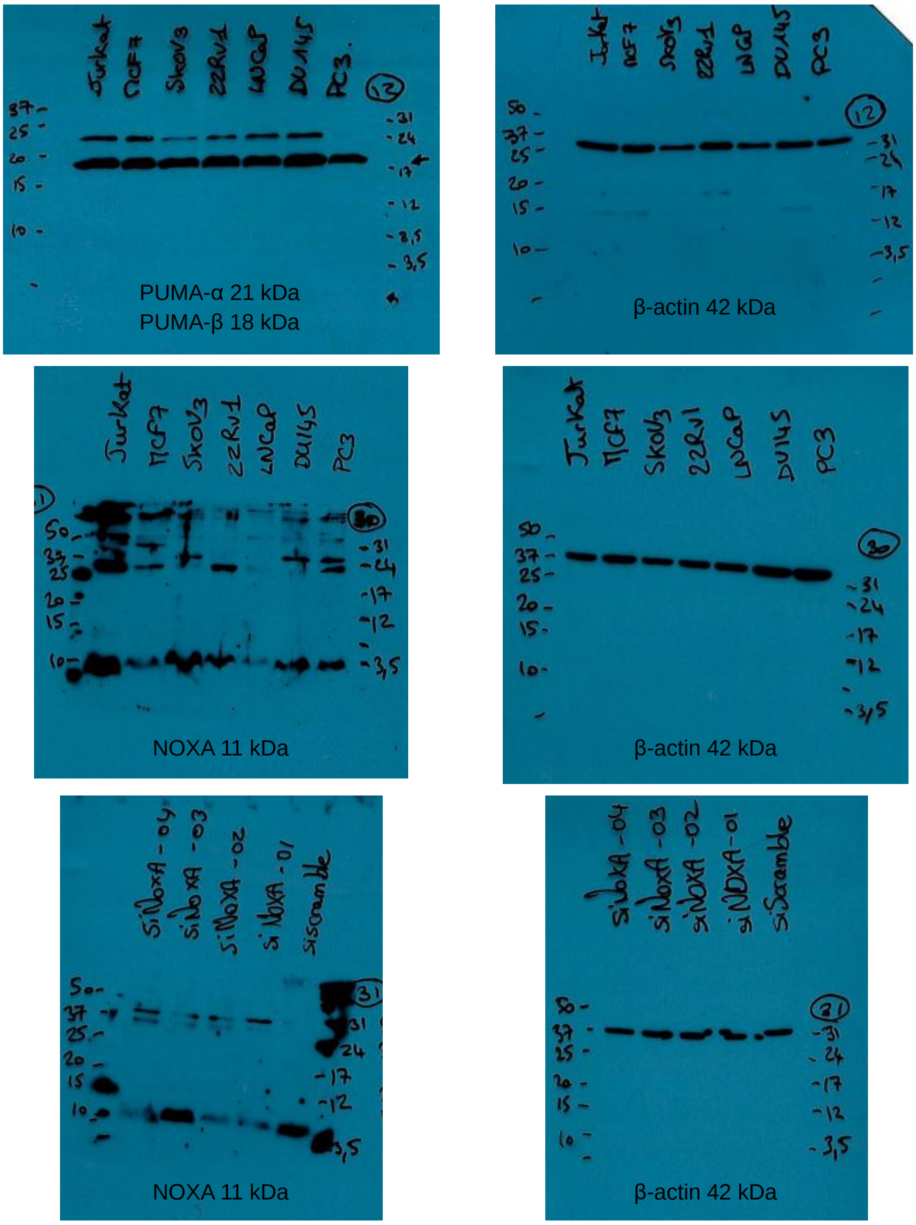

B

	22Rv1	LNCaP	DU145	PC3	
PUMA-alpha	0.60	1.10	1.46	0.45	
PUMA-beta	1.65	2.45	3.62	2.28	
PUMA-total	2.25	3.55	5.08	2.73	
	22Rv1	LNCaP	DU145	PC3	
NOXA	1.48	0.70	1.82	0.91	
	siScramble	siNOXA-01	siNOXA-02	siNOXA-03	siNOXA-04
NOXA	1.91	0.88	0.88	1.65	0.65

Figure S5. Whole Western blots of PUMA and NOXA expression in PC cell lines. (**A**) Detection of PUMA and NOXA in whole cell lysates in PC cell lines, with β-Actin as a control. (**B**) Intensity ratio of biomarkers for each cell line, normalized with β-Actin.

Table S1. Description of primary antibodies and conditions used for IF.

Markers		Company	Catalog No.	Clone	Ventana Staining		
					Antigen retrieval	Primary Antibody	
					Reagent and Time (min)	Dilution	Time in minutes
PUMA		Abcam	Ab33906	EP512Y	CC2, 60	1:1000	60
NOXA		Thermo Fisher Scientific	MA1-41000	114C307.1	CC1, 60	1:25	60
Epithelial mask	CK8	Thermo Fisher Scientific	MA5-14428	TS1	NA	1:100	60
	CK18	Santa Cruz Biothechnogy	sc-6259	DC-10	NA	1:100	60
	CK8 and 18	DAKO	Flex	clone EP17/30	NA	1:2	60
Basal mask	p63	Neomarkers	Ab-1	4A4	NA	1:200	60
	CK HMW	Cedarlane	CLSG36689-05	34bE12	NA	1:50	60

Abbreviations: CC1/CC2 = cell conditioning 1/2 solution (supplied by Ventana Medical Systems), CK = cytokeratin, HMW = high molecular weight, NA = not applicable.

Table S2. Description of secondary antibodies and conditions used for IF.

Marker	Catalog No.	Condition	Excitation (nm)	Emission (nm)
Cy5™ goat anti-rabbit IgG	A10523	1:250, PBS-BSA 1%	649	666
Cy5™ goat anti-mouse IgG	A10524	1:250, PBS-BSA 1%	649	666
Alexa Fluor® 488 goat anti-mouse IgG	A11001	1:250, PBS-BSA 1%	490	525
Alexa Fluor® 488 goat anti-rabbit IgG	A11008	1:250, PBS-BSA 1%	490	525
Alexa Fluor® 546 donkey anti-mouse IgG	A10036	1:250, PBS-BSA 1%	556	573
DAPI, dilactate	D3571	1:10000, PBS 1X	350	470

Abbreviations: PBS = phosphate-buffered saline, BSA = bovine serum albumin. All antibodies were purchased from Thermo Fisher Scientific.

References

1. Nakano, K.; Vousden, K.H. PUMA, a novel proapoptotic gene, is induced by p53. *Mol. Cell* **2001**, *7*, 683–694.
2. Cazanave, S.C.; Mott, J.L.; Elmi, N.A.; Bronk, S.F.; Werneburg, N.W.; Akazawa, Y.; Kahraman, A.; Garrison, S.P.; Zambetti, G.P.; Charlton, M.R.; et al. JNK1-dependent PUMA expression contributes to hepatocyte lipoapoptosis. *J. Biol. Chem.* **2009**, *284*, 26591–26602.
